# Community Perceptions of Integrating Community Health Workers and Telehealth Services for Chronic Disease Management in a Rural Island Community: A Qualitative Study

**DOI:** 10.2196/86907

**Published:** 2026-03-19

**Authors:** Jose G Perez-Ramos, Scott McIntosh, Joselyn Wei Chen, Jessica Alicea-Vellon, Carlos E Rodriguez-Diaz

**Affiliations:** 1 Department of Public Health Sciences, School of Medicine and Dentistry, University of Rochester, 265 Crittenden Blvd CU420644, Rochester, NY, 14642, United States, 1 5852768755; 2 Health ProMed Inc, San Juan, Puerto Rico; 3 School of Public Health, Boston University, Boston, MA, United States

**Keywords:** telehealth, chronic disease, Puerto Rico, community health workers, public health, community-oriented primary care, COPC

## Abstract

**Background:**

Rural and isolated communities, such as Culebra, Puerto Rico, face significant health care challenges due to geographic isolation, limited medical resources, and socioeconomic disadvantages. Chronic diseases, particularly diabetes and hypertension, are highly prevalent and contribute to poor health outcomes. Telehealth services (THS) and community health workers (CHWs) have been identified as effective interventions for improving health care access in underserved areas. This study is grounded in the principles of community-oriented primary care, which emphasizes the integration of primary care and public health for a defined community, and positions the combination of CHWs and THS as a practical application of this model in an isolated island setting.

**Objective:**

This study aimed to explore community perceptions and attitudes toward integrating THS with the support of CHWs to improve chronic disease management in Culebra, Puerto Rico.

**Methods:**

A qualitative study using semistructured individual interviews was conducted with 20 patients from Culebra’s Federally Qualified Health Center. Interviews were guided by the socioecological model to assess community perspectives at the individual, interpersonal, community, and societal levels. Thematic analysis was conducted using Dedoose by the principal investigator and a research assistant, with coding discrepancies resolved through negotiated agreement. Translation and back-translation of themes followed the Brislin method. The study adhered to the COREQ (Consolidated Criteria for Reporting Qualitative Research) guidelines.

**Results:**

Thematic analysis revealed three primary themes: (1) the pervasive impact of social determinants of health on daily life and health care access, (2) the valued and trusted role of CHWs in the community, and (3) the dual potential and challenges of THS. CHWs were recognized as critical for improving health care access through appointment assistance, medication management, and emotional support. THS was viewed positively for its potential to reduce transportation barriers and improve continuity of care, although concerns regarding technology access, convenience, and data privacy were also raised. The integration of CHWs and THS was broadly viewed as a promising and trustworthy solution to chronic disease management challenges.

**Conclusions:**

This study highlights the potential of an integrated CHW-assisted THS model as a practical application of community-oriented primary care principles in isolated island communities. The combination of CHWs and THS shows promise for reducing health care disparities and improving chronic disease management. Future research should focus on implementing and evaluating this model through participatory approaches, assessing clinical outcomes and cost-effectiveness, while policy efforts should prioritize THS infrastructure investment and standardized CHW training curriculum.

## Introduction

### Background

In the United States, 1 in 5 communities is a rural or isolated area facing numerous health care challenges due to limited access to services, lower socioeconomic status, and geographic isolation from services [1-3]. The health outcomes of people living in rural communities, such as survival rate, complication frequency, and disease management, are often worse compared with those of people living in urban areas, resulting in a significant public health crisis [4]. The same factors impact the health and health care of persons in Latinx communities, including a higher prevalence of chronic conditions such as diabetes (10.3% in Hispanic communities compared with 8.5% in non-Hispanic White communities) among adults aged 18 years or older [5]. These disparities are even more pronounced for people living in Puerto Rico (a US territory), who experience the worst health outcomes compared with the general US population, having an estimated diabetes prevalence of 20.1% among adults, substantially higher than the US average. Rates of hypertension and high cholesterol also exceed those in other jurisdictions [6,7].

Puerto Rico is an archipelago, and its smallest inhabited island municipality, Culebra, exemplifies the challenges of health care delivery in an isolated setting. Located 17 miles off the coast of the main island, Culebra has a population of approximately 1800 residents [8,9]. The island has no hospital; health care infrastructure is limited to an emergency room with minimal resources, 1 private physician’s office, and a single Federally Qualified Health Center (FQHC), HealthproMed, which was the site of this study. In 2019, the estimated prevalence of diabetes and hypertension in Culebra were 15.1% and 39.2%, respectively [10]. While most residents have health insurance, either through the federally funded, government-run *Plan Vital* or through private plans, structural barriers to accessing care remain significant.

These challenges are social determinants of health (SDoH) and are directly associated with the archipelago’s sociopolitical structural status, which results in significant health inequities [11]. In response, innovative models of care are needed. This study is conceptually grounded in the principles of community-oriented primary care (COPC), an approach that originated in South Africa and emphasizes the integration of primary care practice with public health for a defined community [12,13]. COPC models are designed to be responsive to a community’s specific health needs by moving beyond the clinic walls to deliver care. A key component of modern COPC, particularly in resource-limited settings, is the use of community health workers (CHWs) and, increasingly, information and communication technologies to extend the reach of the health care system [14,15].

In Culebra, a single CHW serves the community, providing a mix of home visits, clinic-based support, and remote coordination of social services via phone. The COVID-19 pandemic accelerated the adoption of telehealth services (THS) globally, and based in part on the findings from this study, the FQHC in Culebra has since expanded its use of telehealth for regular patient visits, even extending these services to the neighboring island of Vieques. The integration of a trusted CHW with accessible THS represents a practical application of the COPC model, tailored to address the unique context of Culebra. However, before the broader implementation of such a model, it was crucial to understand the community’s perspective.

### Aims and Objectives

The primary aim of this study was to explore community perceptions and attitudes toward integrating THS with the support of CHWs to improve health care access for people with chronic diseases in the isolated island municipality of Culebra, Puerto Rico.

### Objectives

The specific objectives were as follows:

1. To assess community members’ attitudes and perceptions toward the role of CHWs in their health care
2. To evaluate community members’ attitudes, perceived benefits, and barriers related to the use of THS for chronic disease management
3. To identify and map the perceived impacts, barriers, and facilitators for implementing an integrated CHW-assisted THS model across the different levels of the socioecological model (SEM)

## Methods

### Study Design and Recruitment

This study used semistructured individual interviews to ascertain community perceptions toward THS infrastructures and the role of CHWs in addressing health access for people with chronic diseases. As part of a decade-long community partnership with the local FQHC and community-based organizations, we recruited 20 community members and patients in Culebra, using a purposive sampling methodology and direct recruitment efforts from the on-site CHWs. Participants were required to be older, be aged at least 18 years old, live in Culebra, have a chronic disease diagnosis, and be able to read the information provided in Spanish for informed consent. The exclusion criteria included nonresidents, nonpatients of the local FQHC, and those unable to communicate in Spanish. The study was conducted in Spanish, the official language in Puerto Rico and the primary language of most of Culebra’s residents, and followed COREQ (Consolidated Criteria for Reporting Qualitative Research) guidelines to report qualitative research (Checklist 1) [16].

It is important to note that this qualitative study represents the first phase of a larger, multiphase participatory research project. Subsequent phases involve data collection with other key stakeholders, including health care providers and patients’ family members, the results of which will be reported in future manuscripts.

### Data Collection

An interview guide (Multimedia Appendix 1) was developed using the SEM framework to capture in-depth perceptions of THS and the role of CHWs in the community as an intervention to increase access to health care. The semistructured qualitative interviews were developed by the principal investigator (PI) and research assistant in Spanish [17]. Our research team members (PI and local CHW), who were experienced in qualitative research methodology, conducted all interviews in Spanish. Interviews were conducted between October 2022 and March 2023, lasting up to 1 hour. To ensure a comfortable and private environment, interviews took place in familiar and convenient community settings, including clinic waiting areas and public spaces nearby. With participants’ consent, all interviews were audio-recorded.

### Ethical Considerations

This study was performed in line with the principles of the Declaration of Helsinki and received ethics approval from the University of Rochester Medical Center Institutional Review Board (STUDY00007257). All participants provided verbal informed consent before the interview began. Participants were informed of their right to discontinue participation or skip questions at any point. The study design prioritized participant privacy and data protection. Audio recordings were initially stored on encrypted devices and subsequently uploaded to a secure, Health Insurance Portability and Accountability Act (HIPAA)–compliant cloud system (Box) at the University of Rochester. All potentially identifiable information was removed from transcripts during the data analysis process to preserve participant confidentiality. Participants were compensated with US $10 cash and offered refreshments as an expression of gratitude for their time.

### Data Analysis

Audio-recorded interviews were transcribed and thematically analyzed using Dedoose, a qualitative analysis software that allows for direct coding [18]. The analysis was guided by the SEM [19], which provided a comprehensive understanding of how participants’ attitudes and perceptions were influenced by factors at the individual, interpersonal, community, and societal levels.

Interview data were examined iteratively using thematic analysis for patterns and themes, investigating community attitudes and perceptions toward THS systems and the CHW’s model [20]. To ensure linguistic and cultural validity, themes and supportive quotes were translated and back-translated from Spanish to English by a team of 3 bilingual laboratory members (2 Puerto Ricans and 1 Peruvian) following the Brislin back-translation method [21]. The coding process was conducted by the PI, the CHW, and a research assistant. Coding discrepancies were resolved through a process of negotiated agreement during weekly team meetings, where differing interpretations were discussed until consensus was achieved [22]. The team also debriefed regularly (≥5 times per week) with partners and community members during fieldwork to discuss emerging findings.

## Results

### Overview

**Figure 1.**
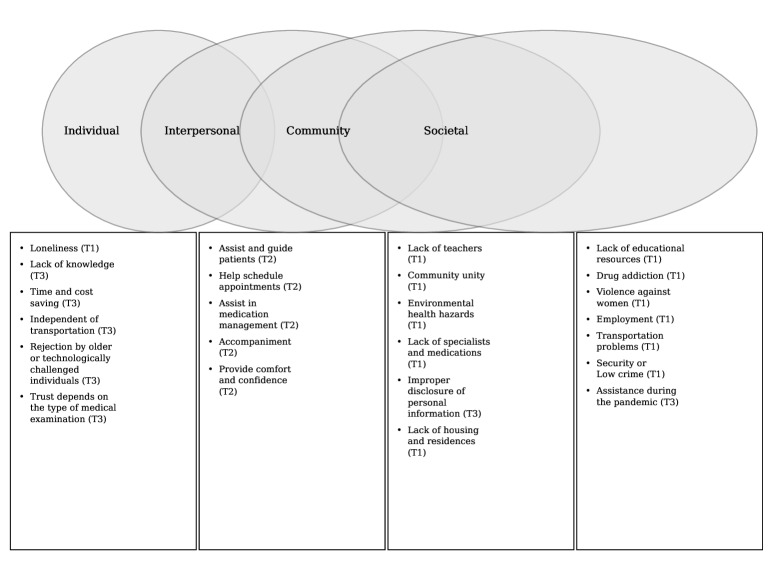
Applied the social determinants of health model to the thematic analysis. T1: theme 1; T2: theme 2; T3: theme 3.

Analysis of the interview data revealed 3 major themes related to the potential integration of CHWs and THS in Culebra: (1) the pervasive impact of SDoH on daily life and health care access, (2) the valued and evolving role of CHWs in the community, and (3) the dual potential and challenges of THS. These themes and their corresponding subthemes were mapped across the 4 levels of the SEM—individual, interpersonal, community, and societal—to provide a comprehensive understanding of the factors influencing community perceptions. As shown in Figure 1, this categorization helped illustrate how these factors interact and contribute to the community’s perspectives on integrating THS with CHW support to improve health care access for people with chronic diseases on the island of Culebra. The most relevant subthemes of each theme will be discussed in the following sections.

In addition, Multimedia Appendix 2 presents the demographic profile of the 20 participants who predominantly self-identified as female (n=12, 60%) and had a diverse age range (20‐70 years). The average interview duration was 55 minutes and 42 seconds (SD 10.8). Participants’ educational levels varied, with 11 (55%) participants having completed or exceeded high school and a quarter (n=5, 25%) having less than a high school education.

### Theme 1: The Pervasive Impact of SDoH on Daily Life and Health Care Access

#### Overview

Participants highlighted several key issues affecting their community, including limited educational resources, economic instability, violence, and inadequate housing and transportation (Multimedia Appendix 3). These factors create significant barriers to health and well-being, emphasizing the need for community-based interventions and policies to address these underlying causes of health disparities in rural and isolated areas.

#### Subtheme: Transportation Problems

Transportation was identified as a significant issue, with the community’s geographical isolation contributing to difficulties in accessing other areas. This affects daily life and service provision. A participant explained, “Well, transportation. Like Vieques and Culebra, we are, as you say, far from the big island, so sometimes we have a lot of problems with transportation, that’s more than most of what it affects” (IND49). This reflects societal-level challenges.

#### Subtheme: Environmental Health Hazards

The improper management of sewage and wastewater was identified as a severe environmental and health concern by the Culebra community. Overflows cause a threat to the cleanliness and hygiene of the community, as expressed by 1 participant: “Sewage is overflowing everywhere. It is really a very strong concern.” On the other hand, participants also discussed the negative impact of exploitation-induced destruction, particularly from investors and unsustainable practices. This leads to the destruction of natural resources and cultural heritage and threatens the community’s long-term well-being. A participant stated, “Our natural resources are being destroyed. The new generations are the ones who are going to have to deal with the exploitation” (IND2). Both issues affect at the community and societal levels.

#### Subtheme: Lack of Medications

A consistent medication shortage was identified as a pressing health issue, particularly affecting emergency services and controlled medications. This hinders the effective management of health conditions. One participant highlighted, “There is a lot of lack of medication. In the emergency area, there is a lot of medication shortage” (IND2). This issue impacts at the individual, community, and societal levels.

#### Subtheme: Lack of Educational Resources

Participants identified a shortage in the availability and quality of educational resources, exemplified by a lack of schools and the underutilization of existing educational spaces. This lack of resources was seen as a barrier to providing adequate education within the community. For instance, 1 participant stated, “Everything that has to do with education, that is what we are looking for” (FG1). These insights reflect significant concerns at the individual, community, and societal levels.

#### Subtheme: Loneliness in Some Individuals

Feelings of isolation and loneliness were identified as significant concerns for specific community members, particularly during times of personal crisis. Loneliness affects mental and emotional well-being. For example, 1 participant noted that some patients like to come to the clinic to have company “because many times they are alone, and what they want is to talk” (IND2). This reflects the individual-level impact of social isolation.

#### Subtheme: Employment

A lack of diverse employment opportunities was highlighted as a significant issue, especially for the youth. The local economy offers limited career paths, forcing individuals to migrate or accept employment outside their desired professions. A participant explained, “For young people, there are not many [options out] there; what we have are the same [jobs] in a restaurant, but there is no way that if someone wants to be a lawyer, there is no way. There are not many alternatives for work” (FG1). The decline in the available labor force, mainly due to an aging population, was seen as a challenge in maintaining essential services. This sense of vulnerability affects those who are aging or ill. This issue impacts the individual, community, and societal levels.

### Theme 2: The Valued and Evolving Role of CHWs

#### Overview

CHWs are vital in enhancing health care access and quality for individuals in underserved communities (Multimedia Appendix 4). They provide essential support by assisting with patient education, scheduling medical appointments, managing medications, and offering companionship during medical visits. Their involvement improves health outcomes and fosters a sense of trust and comfort among patients, significantly impacting their overall health care experience. However, most people do not fully understand CHWs’ role, even though some might already have been in contact with CHWs before. Therefore, it is essential for people to know what CHWs can do for them to improve their health care access.

#### Subtheme: Assists and Guides the Patient

Participants highlighted the vital role of CHWs in providing valuable assistance and guidance. CHWs help patients understand their health conditions and learn how to manage their treatments, improving their autonomy and care. One participant noted, “They guide you, give you information so you know what these symptoms are based on, and how to take care of yourself” (FG103). Another shared, “They have taught me how to inject insulin. I have done it, but I have had several doubts. And they clear up my doubts” (IND39). These interactions enhance the interpersonal relationships between CHWs and patients, fostering trust and confidence.

#### Subtheme: Helps Schedule Appointments With Specialists

CHWs play a crucial role in helping patients schedule appointments with specialists, ensuring continuous and specialized care, especially for chronic conditions. A participant explained, “If I needed such an appointment with the specialist, find me one. When I need a referral, what I do is that I go with the community health worker, we interconnect, and things seem to move a little faster; that’s why it’s important” (IND2). This assistance is essential in maintaining the continuity of care and addressing the specific health needs of patients.

#### Subtheme: Assists in Medication Management

The support provided by CHWs in medication management is seen as crucial, particularly for those who struggle with maintaining their medication regimens. One participant stated simply, “They get the medicines for me” (IND59). This service is essential for ensuring that patients adhere to their prescribed treatments and manage their health conditions effectively.

#### Subtheme: Trust and Social and Health Support

Participants valued the accompaniment provided by CHWs when accessing medical facilities. This support offers comfort and confidence, particularly when patients need to access laboratory services or return home. One participant shared, “And I have a lot of confidence in her when I have to go to the laboratory. She waits for me patiently. She takes me home” (IND59). This accompaniment helps reduce the stress and anxiety associated with medical appointments, contributing to better health outcomes.

#### Subtheme: Provides Comfort and Confidence

CHWs are recognized for their attentive and considerate care, which instills comfort and confidence in patients. As one participant expressed, “They gave me comfort” (IND49). This emotional support is essential to the health care experience, helping patients feel more secure and supported in managing their health.

By offering these services, CHWs play an integral role in improving health care delivery and outcomes, particularly in underserved communities. Their work addresses both the practical and emotional needs of patients, fostering a more supportive and effective health care environment.

### Theme 3: The Dual Potential and Challenges of THS

#### Overview

Participants expressed different perspectives toward the THS in providing health care access in rural and underserved communities for chronic disease management (Multimedia Appendix 5). Some participants believed that THS could offer numerous benefits, including reducing the need for transportation, saving time and costs, and ensuring continuity of care when in-person medical visit options were not available or accessible. However, THS also faces challenges such as perceived inconvenience compared with in-person visits, lack of knowledge among users, and concerns about data privacy. Despite these challenges, THS was seen as a beneficial alternative when traditional health care options are inaccessible, enhancing overall health care experiences by offering peace of mind and improved communication between patients and health care providers.

#### Subtheme: Less Convenient Than In-Person Care

Participants identified concerns about the convenience of remote health care services compared with in-person visits, especially highlighting the value of physical exams and the personal touch of face-to-face consultations. One participant stated, “In-person visits are more convenient” (IND39). This underscores the interpersonal challenge of remote health care services.

#### Subtheme: Assistance During the Pandemic

The crucial role of THS during the pandemic was emphasized, providing necessary support and care in challenging times when traditional services were disrupted. A participant noted, “It was a great moment. And if you come to apply it in the case of Culebra, it would be phenomenal, too. I would want to have a doctor all the time, but if I have no other option, well, it’s great that this option is there” (IND2). This highlights the community and societal benefits of THS during crises.

#### Subtheme: A Good Alternative When No Other Option Is Available

Participants viewed THS services as beneficial alternatives when in-person care is inaccessible, mainly due to transportation issues. One participant shared, “I don’t know about anyone else, but I do. I prefer a doctor on camera than none at all” (IND39). This demonstrates the individual, community, and societal advantages of having THS as an option.

#### Subtheme: Lack of Knowledge

More knowledge about THS and procedures still needs to be provided, which may prevent individuals from fully using them. A participant admitted, “I haven’t done it yet” (IND49). This reflects the individual-level challenge of awareness and education regarding THS.

#### Subtheme: Independent of Transportation

The advantage of THS not requiring transportation was particularly noted as useful for communities with limited access to transportation. One participant explained, “In bad weather that there is no transportation which cannot come, then that would be a good alternative” (IND39). This benefit spans community and societal levels.

#### Subtheme: Time and Cost Savings

Participants identified significant time and cost savings with the use of remote or THS, eliminating the need for travel and reducing the opportunity cost of seeking medical care. One participant stated, “These trips would actually waste money and time” (FG103). Another noted, “It saves a lot of time because the kids don’t have to miss school. It’s a whole lost day” (IND1). These points highlight the individual and community benefits of THS.

#### Subtheme: Privacy Issues—Improper Disclosure of Personal Information

Concerns were raised about the potential for improper disclosure of personal information when using remote services, which may deter some from using these services. A participant expressed, “There are also many people who do not like to give [their] data over the phone. There have been many hackers” (IND49). This concern affects individual, interpersonal, community, and societal levels.

#### Subtheme: Rejection by Older or Technologically Challenged Individuals

Participants identified that older individuals or those who are less technologically savvy may resist using remote health care services due to unfamiliarity with technology or a preference for traditional in-person care. One participant’s opinion was, “Because sometimes there are many elderly people, they don’t know technology” (IND49). This reflects the individual and community challenges in THS adoption.

#### Subtheme: Trust Depends on the Type of Medical Examination

Trust in the THS varied depending on the type of medical examination required. For visual assessments or follow-ups, remote consultations would suffice, but for more tactile, in-depth examinations, in-person visits were preferred to ensure safety and accuracy. One participant remarked, “The decision depends on whether it is a follow-up consultation or if it is the first time with the specialist. If it is an evaluation or, if they need to do a laboratory check, and if they are going to do a study later. Therefore, people should go in person” (FG103). This indicates the individual-level trust considerations.

#### Subtheme: Peace of Mind

Having health care consultations in the comfort of their own home provides patients with peace of mind. It allows them to communicate more effectively, ensuring they remember to discuss all their concerns with the physician. A participant shared, “Yes, I sit in my room and explain everything to him. Sometimes, you go to see the doctor, and you forget the things that you had to tell him” (IND59). This benefit is significant at the individual level.

## Discussion

### Principal Findings

This study aimed to explore community perceptions of an integrated CHW-assisted THS model to improve health care access for people with chronic diseases in the isolated setting of Culebra, Puerto Rico. Our findings suggest a general community acceptance of this model, which can be understood as a modern application of the COPC framework. By integrating clinic-based services with community-based assets—the CHW and technology—the proposed model aligns with the core COPC principle of providing continuous, needs-based care to a defined population [12].

Our findings successfully met the study’s 3 primary objectives. First, we found that the community perceives CHWs as vital, trusted figures who provide essential assistance, guidance, and comfort. This aligns with COPC models that rely on CHWs to build trust and bridge the gap between the formal health system and the community. Second, participants viewed THS as a valuable tool to overcome structural barriers such as transportation, although they also raised concerns about the digital divide and the loss of in-person contact. This highlights a key challenge in implementing information and communication technology–enabled COPC, that is, ensuring technology is deployed equitably. Finally, the SEM framework effectively mapped the complex interplay of barriers and facilitators, confirming that a successful COPC intervention must address challenges at multiple levels, from individual-level trust to societal-level infrastructure.

The findings confirm that while CHWs can help mitigate some SDoH barriers at the individual and interpersonal levels, they cannot resolve larger, structural determinants. However, the integration of CHWs and THS was seen as a powerful combination. This synergy, a hallmark of contemporary COPC, can help bridge gaps in care by leveraging technology to bring specialists to the island virtually, while relying on the personalized support of CHWs to facilitate these interactions. This aligns with existing literature demonstrating the effectiveness of CHWs in navigating services for Latinx communities and the power of THS for providing routine care leading to better health outcomes, although few studies have explored their combined impact within a COPC framework [23].

By adopting the evidence-based models of CHWs and THS, rural island communities can improve health care access, enhance health education, and build sustainable health care systems. This can lead to better health outcomes and reduced health disparities, ultimately benefiting communities such as Culebra and similar rural island populations globally.

### Study Limitations

This study has several limitations inherent to its qualitative design. First, the findings are based on a small, purposive sample from a single, unique island community. Therefore, the results may not be generalizable but offer deep, context-specific insights that can be instrumental in adapting for future studies of similar island communities. Second, as the data are self-reported, they are subject to participants’ personal biases. Third, the research team included the local CHW, which may have introduced a degree of social desirability bias, although this relationship also facilitated trust. We sought to mitigate this by using a neutral, university-affiliated PI and a research assistant from another country and with limited knowledge of Puerto Rico. Finally, while this initial phase did not involve direct co-design of research questions with community members, the questions were reviewed and refined in collaboration with the local FQHC—an institution that represents and is embedded in the local community’s health care perspectives and needs. This study is the first phase of a larger participatory project in which subsequent phases will engage patients, families, and health care providers more directly.

## Supplementary material

10.2196/86907 Multimedia Appendix 1 Sample interview guide questions.

10.2196/86907 Multimedia Appendix 2 Key descriptive information of the interview sample (N=20), including gender distribution, average interview duration, age groups, and educational attainment. Participants included 12 women (60%) and 8 men (40%), with interviews lasting an average of 55 minutes and 42 seconds. The sample represented a range of age groups (N=20), with the largest proportion of participants not disclosing their age (n=8, 40%), followed by individuals in their 50s (n=3, 15%), and other adult age categories. Educational attainment also varied across participants, with most reporting high school completion (n=9, 45%), followed by less than high school (n=5, 25%), postsecondary education (n=2, 10%), and a proportion who did not disclose their educational level (n=4, 20%).

10.2196/86907 Multimedia Appendix 3 Subthemes emerging from theme 1—the pervasive impact of social determinants of health on daily life and health care access—organized across the 4 domains of the socioecological model (individual, interpersonal, community, and societal). The table illustrates how structural and environmental factors such as lack of educational resources, employment challenges, housing insecurity, and limited medication access manifest at multiple ecological levels to shape health outcomes and health care use.

10.2196/86907 Multimedia Appendix 4 Subthemes emerging from theme 2—the valued and evolving role of community health workers—mapped across the 4 domains of the socioecological model. The table highlights how community health workers contribute across individual and interpersonal levels by assisting with appointment scheduling, medication management, and emotional support, while also providing comfort and confidence at the community and societal levels.

10.2196/86907 Multimedia Appendix 5 Subthemes emerging from theme 3—the dual potential and challenges of telehealth services—distributed across the 4 domains of the socioecological model. The table captures both the perceived benefits of telehealth (eg, cost savings, transportation independence, and pandemic-era access) and its limitations (eg, privacy concerns, rejection by older or technologically challenged individuals, and reduced convenience compared with in-person care) as experienced across individual, interpersonal, community, and societal domains.

10.2196/86907 Checklist 1 COREQ checklist.

## Abbreviations

CHW: community health worker
COPC: community-oriented primary care
COREQ: Consolidated Criteria for Reporting Qualitative Research
FQHC: Federally Qualified Health Center
HIPAA: Health Insurance Portability and Accountability Act
PI: principal investigator
SDoH: social determinants of health
SEM: socioecological model
THS: telehealth services
