# Supplementary material for: Community Perceptions of Integrating Community Health Workers and Telehealth Services for Chronic Disease Management in a Rural Island Community: A Qualitative Study
Source: J Particip Med. 2026 Mar 19;18:e86907. doi: 10.2196/86907 (PMC13002157; doi:10.2196/86907)
Supplement: Multimedia Appendix 1 [file jopm-v18-e86907-s001.docx]

Appendix A: Sample Interview Guide Questions and Socio-Ecological Model (SEM) Match across domains

This guide was developed using the Socio-Ecological Model (SEM) to frame questions across different domains. The following are sample questions used to guide the semi-structured interviews.

Introduction & Consent

- The interviewer introduces themselves and the study purpose.
- Confidentiality and the right to withdraw are explained.
- Verbal consent to participate and be audio-recorded is obtained.

Domain 1: Community Context

- "Please tell us about your community here. What is it like?"
- "What are the challenges facing your community? (Social, environmental)"
- "What are the strengths of your community?"
- "Please tell us what it is like to live here day-to-day."

Domain 2: Health and Healthcare Access

- "Please tell us about the health care system here – clinics, doctors, etc."
- "What health concerns do you have in your community?"
- "What difficulties have you faced taking care of your health conditions?"
- "Can you give us examples of how you manage your health care needs?"

Domain 3: Perceptions of Telehealth Services (THS)

- "How would you feel interacting with a health care provider via a computer or phone instead of an in-person visit?"
- "What do you know about Telehealth? What is it for?"
- "What do you think is the biggest benefit of telehealth in general?"
- "Have you ever used it? If yes, can you tell us about your experience? If no, why not?"
- "Is Telehealth more convenient than in-person visits? Why or why not?"
- "Why do you think some people will not use Telehealth?"

Domain 4: Perceptions of Community Health Workers (CHWs)

- "Please describe how a community health worker can be helpful for the community."
- "How would you feel interacting with a community health worker to help manage your health care and social needs?"
- "Have you had an interaction with a community health worker before? How was your experience?"
- "If you have not, would you try it? How would you benefit from having a community health worker?"

Closing

- "What are the greatest assets of your community?"
- "What do you like the most about your community?"
- "Thank you for your time."
