# Supplementary figures and images for: Community Perceptions of Integrating Community Health Workers and Telehealth Services for Chronic Disease Management in a Rural Island Community: A Qualitative Study
Source: J Particip Med. 2026 Mar 19;18:e86907. doi: 10.2196/86907 (PMC13002157; doi:10.2196/86907)

Table 1- Qualitative Research Logistic and Demographic Information


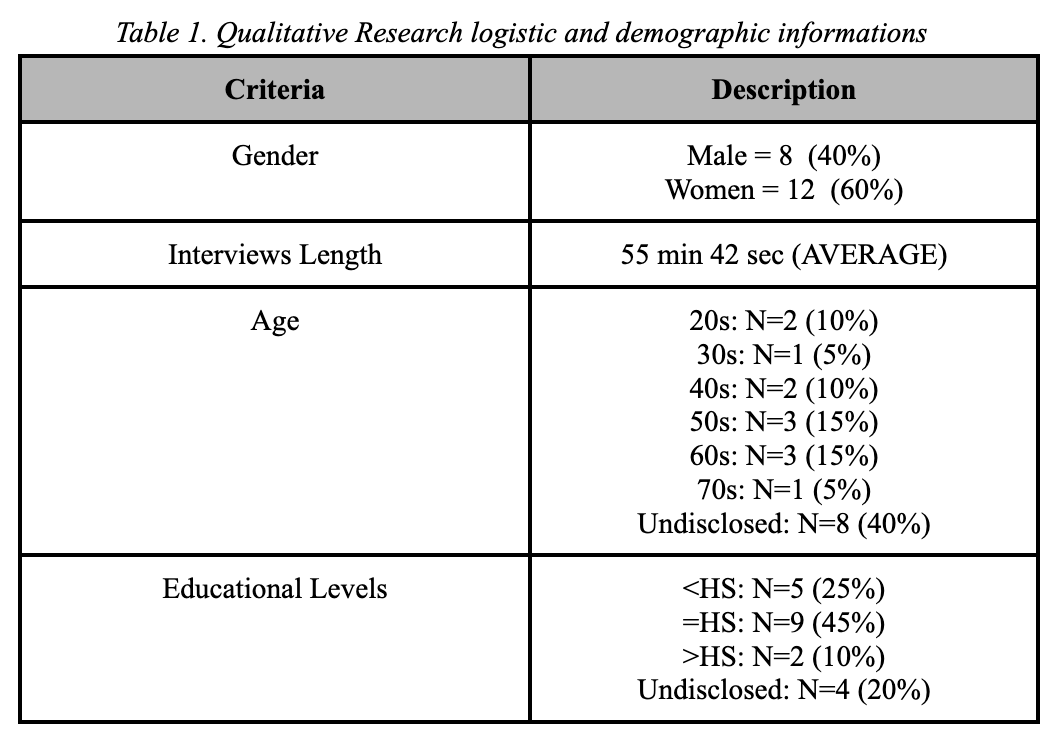

Supplement: Multimedia Appendix 2 [file jopm-v18-e86907-s002.docx]
