# Supplementary material for: Community Perceptions of Integrating Community Health Workers and Telehealth Services for Chronic Disease Management in a Rural Island Community: A Qualitative Study
Source: J Particip Med. 2026 Mar 19;18:e86907. doi: 10.2196/86907 (PMC13002157; doi:10.2196/86907)
Supplement: Multimedia Appendix 3 [file jopm-v18-e86907-s003.docx]

**Table 2: Theme 1 - *The Pervasive Impact of Social Determinants of Health on the Daily Life and Healthcare Access***

| **Domains** | **Individual** | **Interpersonal** | **Community** | **Societal** |
| --- | --- | --- | --- | --- |
| **Subthemes** | - Lack of education resources - Loneliness - Employment - Lack of housing and residences - Lack of medications | Employment | - Lack of education resources - Employment - Community unity - Environmental health hazards - Lack of medications | - Lack of education resources - Employment - Lack of housing and residences - Transportation problems - Safety/Low crime - Community unity - Environmental health hazards - Lack of medications |
