# Supplementary material for: Community Perceptions of Integrating Community Health Workers and Telehealth Services for Chronic Disease Management in a Rural Island Community: A Qualitative Study
Source: J Particip Med. 2026 Mar 19;18:e86907. doi: 10.2196/86907 (PMC13002157; doi:10.2196/86907)
Supplement: Multimedia Appendix 4 [file jopm-v18-e86907-s004.docx]

**Table 3: Theme 2 – The Valued and Evolving Role of Community Health Workers**

| **Domains** | **Individual** | **Interpersonal** | **Community** | **Societal** |
| --- | --- | --- | --- | --- |
| **Subthemes** | - Helps schedule appointments with specialists - Assists in medication management - Provides comfort and confidence | - Assist and guides the patient - Helps schedule appointments with specialists - Assists in medication management - Trust and social/health support - Provides comfort and confidence | - Provides comfort and confidence | - Provides comfort and confidence |
