# Supplementary material for: Community Perceptions of Integrating Community Health Workers and Telehealth Services for Chronic Disease Management in a Rural Island Community: A Qualitative Study
Source: J Particip Med. 2026 Mar 19;18:e86907. doi: 10.2196/86907 (PMC13002157; doi:10.2196/86907)
Supplement: Multimedia Appendix 5 [file jopm-v18-e86907-s005.docx]

**Table 4: Theme 3 – The Dual Potential and Challenges of Telehealth Services**

| **Domains** | **Individual** | **Interpersonal** | **Community** | **Societal** |
| --- | --- | --- | --- | --- |
| **Subthemes** | - A good alternative when no other option is available - Lack of knowledge - Time and cost saving - Possible improper disclosure of personal information - Rejection by older or technologically challenged individuals - Trust depends on the type of medical examination - Peace of Mind | - Less convenient than in-person care - Possible improper disclosure of personal information | - Assistance during the pandemic - A good alternative when no other option is available - Independent of transportation - Time and cost saving - Possible improper disclosure of personal information - Rejection by older or technologically challenged individuals | - Assistance during the pandemic - A good alternative when no other option is available - Independent of transportation - Possible improper disclosure of personal information |
