# Supplementary material for: Community Perceptions of Integrating Community Health Workers and Telehealth Services for Chronic Disease Management in a Rural Island Community: A Qualitative Study
Source: J Particip Med. 2026 Mar 19;18:e86907. doi: 10.2196/86907 (PMC13002157; doi:10.2196/86907)
Supplement: Checklist 1 [file jopm-v18-e86907-s006.pdf]

Supplementary File 1: COREQ Checklist

Consolidated Criteria for Reporting Qualitative Research (COREQ): 32-item checklist

This checklist is based on the COREQ guidelines and indicates where in the manuscript each item is addressed.

No.	Item	Guide Questions	Reported on Page(s)
**Domain 1: Research Team and Reflexivity**			
1	Interviewer/Facilitator	Which author/s conducted the interview or focus group?	Methods
2	Credentials	What were the researcher's credentials?	Methods
3	Occupation	What was their occupation at the time of the study?	Methods
4	Gender	Was the researcher male or female?	Methods
5	Experience and Training	What experience or training did the researcher have?	Methods
6	Relationship with Participants	Was a relationship established prior to study commencement?	Methods
7	Participant Knowledge of the Interviewer	What did the participants know about the researcher?	Methods
8	Interviewer Characteristics	What characteristics were reported about the interviewer/facilitator?	Methods
**Domain 2: Study Design**			
9	Methodological Orientation	What methodological orientation was stated to underpin the study?	Methods
10	Sampling	How were participants selected?	Methods
11	Method of Approach	How were participants approached?	Methods

No.	Item	Guide Questions	Reported on Page(s)
12	Sample Size	How many participants were in the study?	Methods
13	Non-participation	How many people refused to participate or dropped out?	N/A
14	Setting of Data Collection	Where was the data collected?	Methods
15	Presence of Non-participants	Was anyone else present besides the participants and researchers?	Methods
16	Description of Sample	What are the important characteristics of the sample?	Table 1
17	Interview Guide	Were questions, prompts, guides provided by the authors?	Appendix A
18	Repeat Interviews	Were repeat interviews carried out?	N/A
19	Audio/Visual Recording	Did the research use audio or visual recording to collect the data?	Methods
20	Field Notes	Were field notes made during and/or after the interview?	Methods
21	Duration	What was the duration of the interviews or focus group?	Methods
22	Data Saturation	Was data saturation discussed?	Methods
23	Transcripts Returned	Were transcripts returned to participants for comment and/or correction?	N/A
**Domain 3: Analysis and Findings**			
24	Number of Data Coders	How many data coders coded the data?	Methods
25	Description of the Coding Tree	Did authors provide a description of the coding tree?	Not Reported
26	Derivation of Themes	Were themes identified in advance or derived from the data?	Methods

No.	Item	Guide Questions	Reported on Page(s)
27	Software	What software, if applicable, was used to manage the data?	Methods
28	Participant Checking	Did participants provide feedback on the findings?	Results
29	Major Themes	Are major themes clearly presented in the findings?	Results
30	Minor Themes	Is there a description of diverse cases or discussion of minor themes?	Results
31	Quotations Presented	Are quotations presented to illustrate the themes/findings?	Results
32	Data and Findings Consistent	Is there consistency between the data presented and the findings?	Results/Discussion
